# An Unusual Presentation of Paget-Schroetter Syndrome: A Reminder on the Importance of Clinical Judgment

**DOI:** 10.7759/cureus.93286

**Published:** 2025-09-26

**Authors:** Ayush Isaac

**Affiliations:** 1 Emergency Care Medicine, Aneurin Bevan University Health Board, Cwmbran, GBR

**Keywords:** clinical judgement, d-dimer, paget-schroetter syndrome, thoracic outlet compression, thrombosis, venous thoracic outlet syndrome

## Abstract

We discuss the case of a 36-year-old woman who presented with persistent right upper limb swelling and pain. A previous negative D-dimer (434 µg/L (normal range <500 µg/L)) resulted in an inappropriate discharge, despite a significant past medical history. The patient was sent for a right upper limb ultrasound Doppler scan due to persistent pain, and a clot in the right supraclavicular subclavian vein was observed, confirming the diagnosis of Paget-Schroetter syndrome (PSS). The clot was successfully removed under the vascular team in Cardiff without any post-op complications. The literature review demonstrates that PSS should be suspected mostly on a clinical basis; the literature does not support the use of a D-dimer to support a suspected upper limb deep vein thrombosis (DVT), as this could delay vital treatment for the patient, as demonstrated in this case.

## Introduction

We discuss the case of a right upper extremity deep vein thrombosis (DVT), or Paget-Schroetter syndrome (PSS). PSS is considered an effort thrombosis of the axillary and/or subclavian vein and is the venous manifestation of thoracic outlet compression syndrome (TOCS). It is a rare condition (incidence of 1-2 per 100,000 per annum) that typically occurs in otherwise young, healthy individuals whose daily routines include repetitive movements of the upper limbs, specifically hyperabduction and extension. This condition is commonly seen in young athletes, such as weightlifters and swimmers [1].

Theory suggests that repeated movement of the upper limbs causes microtrauma to the veins, which damages the endothelium, which in turn causes clot formation in these large vessels. Furthermore, anatomical abnormalities of the thoracic outlet narrow the diameter of the vessel, causing blood stasis, which in turn exacerbates clot formation [2].

Although a rare condition, it is an important differential for healthcare professionals not to miss, especially as the consequences can be debilitating. There are a few reasons why this condition can be missed; firstly, the condition is so rare that many physicians may not be familiar with this pathology; secondly, the condition can present with vague symptoms of simply swelling and discolouration, which makes it challenging diagnostically; and finally, the young and fit demographic that typically presents with this problem may lead the clinician to conclude that the cause of their symptoms is related to another system, such as musculoskeletal. Furthermore, management for this condition includes thrombolysis and surgical intervention, unlike a lower limb DVT, which is typically managed with anticoagulation alone for three to six months.

This case will look at the presentation of a young, healthy patient with a previous occurrence of PSS in her left upper extremity, but otherwise no risk factors for this specific condition. This case report is intended to highlight the importance of not relying solely on biochemical markers (in this scenario, a D-dimer blood test) to rule out a pathology if the clinical presentation suggests otherwise.

## Case presentation

We present the case of a 36-year-old woman who presented to the emergency department (ED) with a one-day history of heaviness and discomfort in her right arm and shoulder. She was clinically stable, with no shortness of breath or chest pain. She did not report any paraesthesias in the affected limb. Her past medical history included anxiety, asthma, one previous emergency C-section, one previous appendicectomy and a previously recorded left thoracic outlet syndrome (PSS) in 2016, for which she underwent thrombolysis and thoracic outlet decompression (TOD) with no post-op complications. Her social history was mostly unremarkable; there was no evidence that she engaged in activities that included repetitive upper limb movements.

Physical findings in the ED revealed redness across her right arm, no swelling, no temperature on palpation of the arm, no neurological deficits and a good range of movement of all joints in the arm. The patient’s chest, abdomen and lower limbs were unremarkable on examination.

Bloods (including a full blood count, liver function test, bone profile, urea and electrolytes, and a D-dimer) and an ECG were ordered, and analgesia was prescribed for the patient. The bloods (Table 1) and the ECG were unremarkable. The D-dimer was 434 µg/L (normal range <500 µg/L). The patient was discharged with safety net advice and instructed when to return should any red-flag symptoms occur.

**Table 1:** Blood tests collected at the first presentation. Blood tests collected in the Accident & Emergency (A&E) department at the first presentation were unremarkable. Notably, the D-dimer was negative.

Blood Test	Value	Normal Range
White cell count	9.7	4.0-11.0 x10^9^/L
Haemoglobin	150	115-165 g/L
Platelets	304	150-400 x10^9^/L
Mean cell volume	101	80-100 fL
Neutrophils	6	1.7-7.5 x10^9^/L
Lymphocytes	2.9	1.0-4.5 x10^9^/L
Eosinophils	0.2	0.0-0.4 x10^9^/L
Sodium	138	133-146 mmol/L
Potassium	3.7	3.5-5.0 mmol/L
Urea	2.9	2.5-7.8 mmol/L
Creatinine	65	mL/min/1.73m^2^
Bilirubin	7	<21 μmol/L
Protein	81	65-85 g/L
Albumin	46	35-50 g/L
Globulin	35	22-43 g/L
Alkaline phosphatase	120	30-130 U/L
Alanine transaminase	19	<41 U/L
C-reactive protein	<10	<10 mg/L
D-dimer	434	<500 μg/L
Prothrombin time	9.7	9.0-12.0 sec
Activated partial thromboplastin time	22.3	20.0-30.0 sec
Fibrinogen	4.5	2.0-4.0 g/L

**Figure 1:**
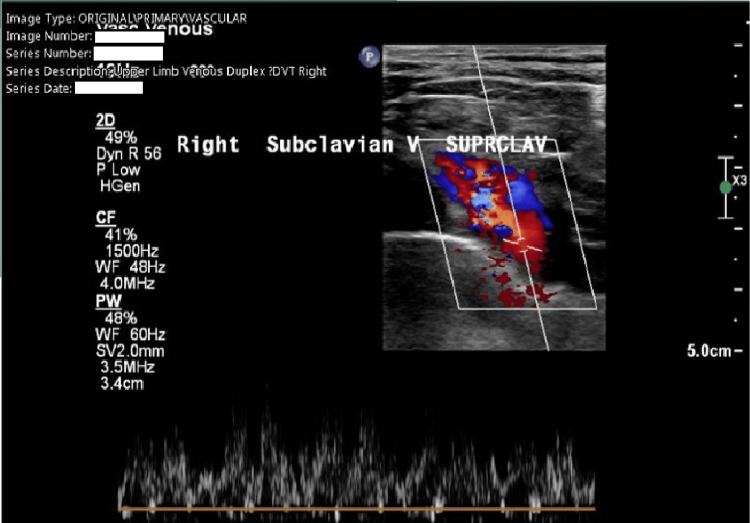
Ultrasound scan of the right upper limb showing the supraclavicular subclavian vein with an echogenic thrombus, as reported by the ultrasound technician.

Two days later, the patient was seen by the general practitioner (GP) with worsening pain and increased swelling in the right arm. The patient was referred to the DVT clinic, where an ultrasound Doppler scan of the right upper extremity was ordered. The right arm ultrasound Doppler scan identified a supraclavicular subclavian vein thrombosis (see Figure 1). At this stage, the patient had been suffering from these symptoms for five days. She was referred on the same day to the Same Day Emergency Care Unit (SDEC) at her local hospital.

On asking the patient, she confirmed that the swelling had deteriorated and she was now experiencing pins and needles. She was otherwise clinically stable, with no chest pain or shortness of breath. On examination, her chest, abdomen and lower limbs were unremarkable. Examination of her right arm revealed gross swelling extending from her hands to her shoulder, with firm swelling noted mainly around the forearm. The radial pulse was palpable bilaterally but was noticeably weaker on her right wrist. Allen’s test was negative. No superficial veins were observed on her neck or chest. A point of care blood test (which included an initial white cell count, haemoglobin, neutrophils and lymphocyte count, sodium, potassium and creatinine), a formal full blood count, urea and electrolyte, C-reactive protein, liver function and bone profile tests were all normal.

She was promptly discussed with the vascular surgical team, as there was concern she had developed PSS in her right upper limb. She was accepted and transferred. She was started on treatment with enoxaparin. An elective thrombolysis and trans-axillary TOD procedure was planned for a later date. Bloods to rule out clotting disorders were completed, which all came back negative (Table 2).

**Table 2:** Clotting screen results performed by the vascular team. All results were within normal limits. The APTT was slightly elevated, likely due to initiation of enoxaparin therapy.

Blood Test	Value	Normal Range
Prothrombin time	10.7	9.0-12.0 sec
Activated partial thromboplastin time (APTT)	41	20.0-30.0 sec
Fibrinogen	4.1	2.0-4.0 g/L
International normalised ratio (INR)	1	Ratio
Protein S assay	92.5	63.0-132.0 IU/dL
Protein C assay	135	70.0-151.0 IU/dL
Factor VIII	131.3	50.0-150.0 IU/dL
IgG anti-cardiolipin antibodies	4.4	0.0-10.0 GPL U/mL
IgM anti-cardiolipin antibodies	2.5	0.0-7.0 MPL U/mL
IgG anti-β2 glycoprotein 1 (B2-GP1) antibodies	3.1	0.0-5.0 U/mL
IgM anti-β2 glycoprotein 1 (B2-GP1) antibodies	2.7	0.0-5.0 U/mL

**Figure 2:**
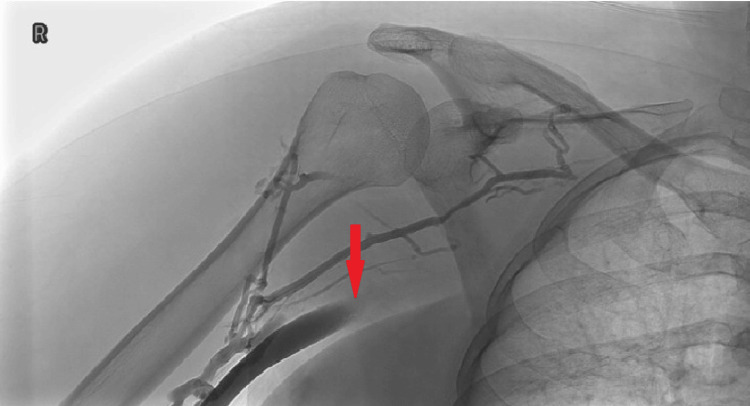
Venogram of the right subclavian vein showing a 3-4 cm central occlusion consistent with thoracic outlet compression. The red arrow indicates the site of the occlusion.

Eleven days from symptom onset, she was admitted to the vascular ward for pulse spray thrombolysis. The venogram (Figure 2) demonstrates a 3-4cm occlusion of the right subclavian vein centrally. Local thrombolysis was performed at the site of the clot, and the catheter remained for an overnight alteplase infusion. The patient was then switched to IV heparin as a bridge to her upcoming TOD surgery.

**Figure 3:**
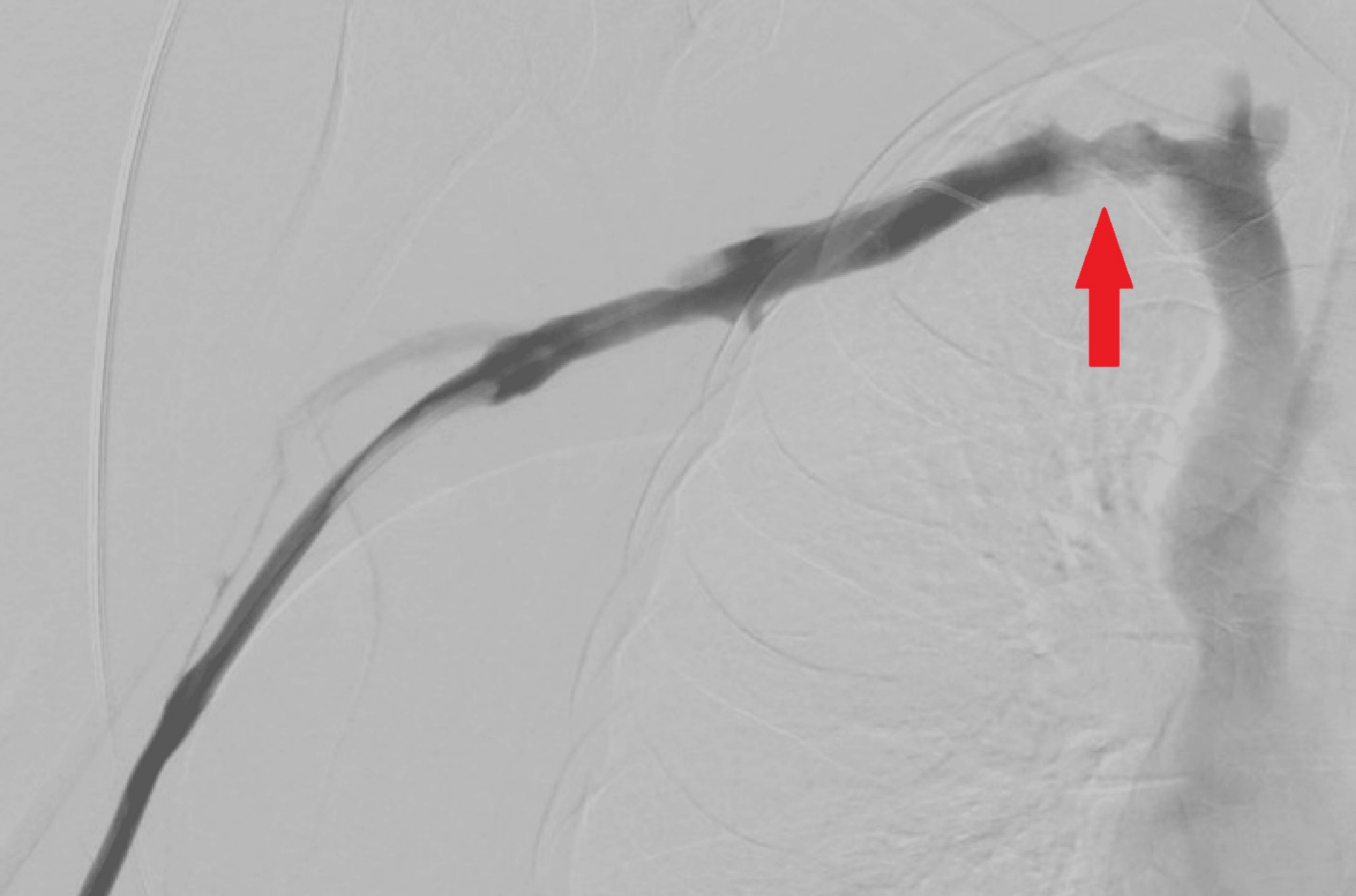
Venogram of the right subclavian vein following overnight tPA infusion. The image demonstrates patency of the subclavian vein, with subclavian notching (red arrow) indicating that the right first rib contributes to thoracic outlet compression. tPA: tissue plasminogen activator

The following day, a repeat venogram was performed, which showed a partially successful thrombolysis procedure, with some subclavian notching, indicating thoracic outlet compression (see Figure 3). The patient continued to be an inpatient until her operation seven days post-thrombolysis. The patient underwent a trans-axillary TOD procedure, and venoplasty was performed to ensure patency of the subclavian vein.

The operation proceeded as follows: a horizontal incision was made in the right axilla, where the costo-brachial nerve was observed and protected. The subclavius and anterior scalene muscles were incised, and the first rib was removed. A chest drain was inserted. Following this, a 7 French (7Fr) sheath was introduced into the basilic vein, and the wire was directed towards the initial subclavian vein stenosis. An 8x40mm ATLAS balloon catheter (Bard Peripheral Vascular, Tempe, AZ, USA) was inflated to maintain patency with a good result.

**Figure 4:**
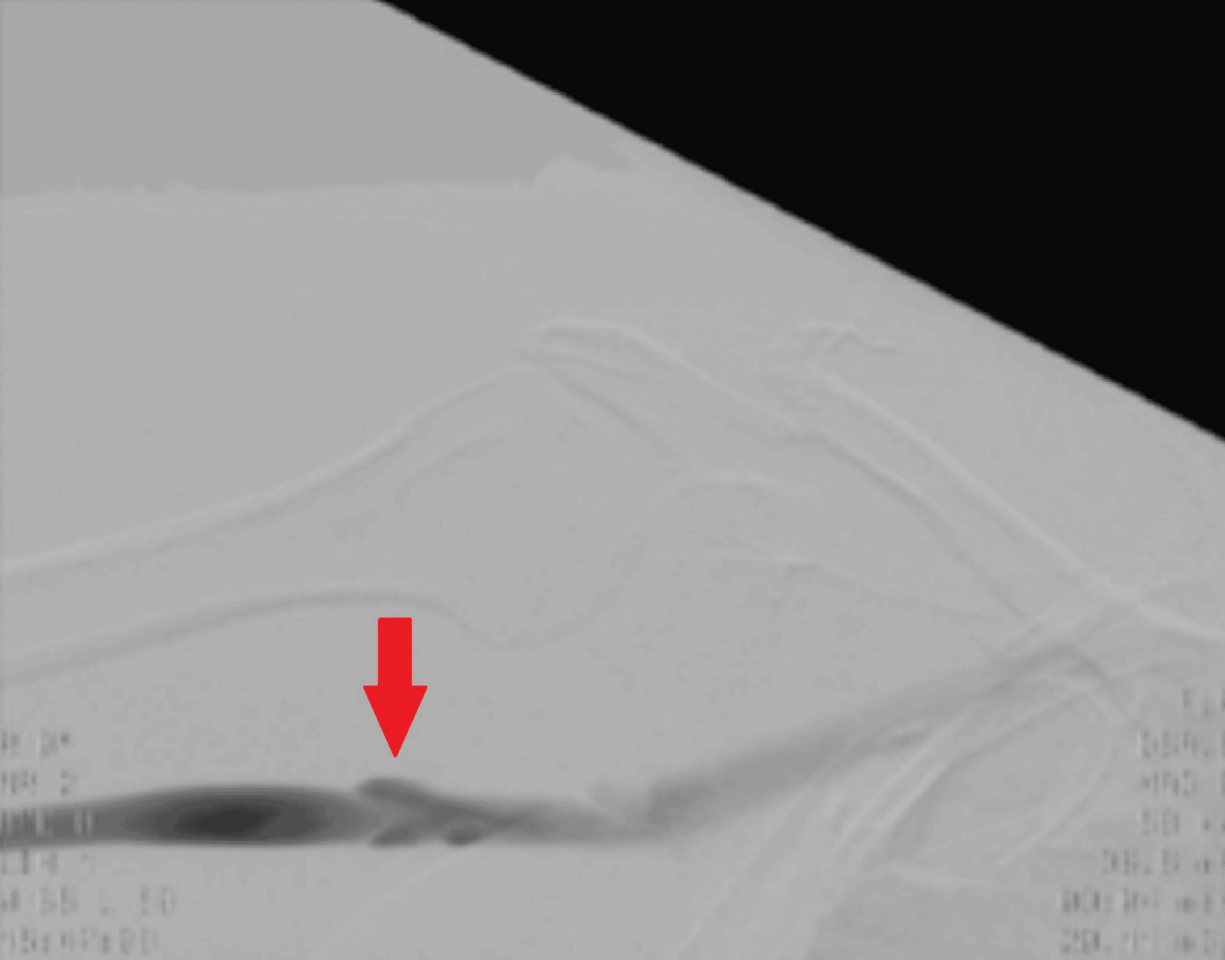
Venogram of the right subclavian vein following venoplasty. The red arrow indicates the site of venoplasty, demonstrating successful restoration of vein patency.

The venogram in Figure 4 demonstrated a good result peri-operatively.

The patient was successfully treated for PSS of her right upper limb (namely, for a venous thrombosis in her right subclavian vein). The patient was admitted under the vascular surgical team for two nights following her operation and discharged. The patient recovered uneventfully, with no neurovascular compromise or recurrence of thrombosis noted during follow-up. She was advised to take apixaban for six months with a follow-up appointment with the vascular surgeon in six weeks.

The patient was also referred to the Haematology Clinic for review of her recurrent PSS. An anti-phospholipid screen was negative, and she was instructed to continue lifelong anticoagulation with apixaban at a reduced dose of 2.5mg twice daily.

## Discussion

As alluded to earlier, PSS or subclavian-axillary vein thrombosis is a subgroup of TOCS, specifically the venous manifestation of TOCS, where the axillary and subclavian veins are the usual suspects for thrombosis of this nature. Subclavian-axillary vein thrombosis is split into two groups: primary and secondary. Primary subclavian-axillary vein thrombosis is caused by anatomical abnormalities and repetitive motions. Secondary subclavian-axillary vein thrombosis is typically caused by trauma from peripherally inserted central lines and thrombophilic conditions, such as factor V Leiden thrombophilia [3,4].

Knowledge of the anatomy of the structures surrounding the arteries, veins and nerves is important for the surgical management of this condition. The thoracic outlet is characterised by three spaces: the interscalene triangle, the costoclavicular space and the retropectoralis minor space. Abnormalities in the scalene muscle and costoclavicular ligaments, as well as osseous malformations (including bone metastases), can contribute to thoracic outlet syndrome [5].

The classical presentation for PSS includes a swollen upper extremity, heaviness or discomfort, pain and paraesthesias. Other symptoms include erythema of the limb and a blue discolouration. Red flags or symptoms that should raise suspicion on first presentation for an upper limb DVT include sudden onset limb pain in the absence of trauma, a young patient, an activity the patient does which includes repetitive upper limb movement and if the patient is unable to elevate their arm without increased pain or paraesthesia [4]. Furthermore, in this case presentation, a previous upper limb DVT should also raise suspicion for recurrence of PSS.

It is important to consider alternative diagnoses, such as trauma, superficial phlebitis, haematomas, etc., as well as life-threatening conditions, such as an atypical presentation of acute coronary syndrome, especially as upper limb DVTs can present as a set of vague symptoms [6,7]. Accordingly, blood tests including full blood count, urea and electrolytes, coagulation, CRP and D-dimer should be performed. Troponin should be considered if the patient has relevant symptoms and medical history. It is vital to obtain an occupational history and ascertain whether there has been any evidence of trauma, as this can contribute towards a diagnosis of venous thoracic outlet syndrome.

Diagnosis is typically confirmed on ultrasound, although a venogram is the gold standard and therefore diagnostic of this particular condition [4]. Ultrasound of the upper limb should not be delayed if there is a strong clinical suspicion of an upper limb DVT. In this patient, diagnosis was delayed by four days (from the ED discharge to the ultrasound scan (USS) of the right arm, confirming the presence of a thrombus in the subclavian vein). On further exploration of the patient’s medical records and results, it was observed that on the patient’s admission for PSS in her left subclavian vein in 2016, the D-dimer prior to this was also negative, thus delaying treatment on her first presentation in 2016. This case underscores the importance of integrating clinical judgement with diagnostic findings to guide timely and effective intervention. According to an article in 2017, D-dimer sensitivities for modern assays range from 75% to 96% [8].

Medical textbooks do not advocate for the use of the D-dimer test as a formal investigation to aid in the diagnosis of PSS - emphasis is put on history and clinical examination, and if there is clinical suspicion of a DVT, a USS should be requested to rule in a thrombus in the upper limb and chest wall vasculature [4,9]. Although D-dimers are a useful tool in the ED, they should not always be used as a replacement for clinical judgement. A recent case report highlights the importance of clinical diligence, where a patient with persistent exertional dyspnoea and pleuritic chest pain with normal biochemical markers (including a negative D-dimer) was eventually imaged and was found to have a pulmonary embolism (PE) [10].

Historically, management for this condition was similar to a lower limb DVT: rest and elevation with anticoagulation. However, the associated comorbidity was high with this method. Modern management typically includes local thrombolysis and TOD, which involves resecting the first rib and any associated structures contributing to the thoracic outlet compression [4].

Clot dissolution is significantly less effective when thrombolysis is delayed beyond 10 days from symptom onset [4]. In this case, the patient received thrombolysis within that window, resulting in partial restoration of vessel patency; this highlights the importance of valuing clinical judgement over investigations and timely intervention for improved patient outcomes.

Previously published case reports describe patients who fit the typical demographic for this condition: young and active, with a history of regular weightlifting, leading to the effort thrombosis known as PSS [6,11]. Another case report described a young patient who did not have an active lifestyle but was on an oral contraceptive, which increased her risk of blood clots [12]. Unlike these patients, the patient presented in this case report did not have any obvious risk factors for her to develop PSS twice.

## Conclusions

To conclude, this case reinforces that in suspected venous thoracic outlet syndrome, a negative D-dimer should not delay imaging and treatment. It is important to remember this condition as a differential for atraumatic upper limb swelling and pain. This case also serves as a reminder to not delay imaging or treatment for other thrombotic conditions, such as lower limb DVTs and PEs; if there is clinical concern for these conditions, imaging should be ordered to confirm a diagnosis, rather than ordering a D-dimer to rule out the pathology. Delays in confirming the diagnosis due to an over-dependence on biochemical markers can contribute to less desirable patient outcomes and potentially affect patient safety as a consequence.
